# Coexistence of invasive ductal breast carcinoma and fibroadenoma

**DOI:** 10.11604/pamj.2019.33.139.17140

**Published:** 2019-06-25

**Authors:** Fatma Saadallah, Imen Bouraoui, Lamia Naija, Saida Sakhri, Ines Zemni, Jamel Ben Hassouna, Tarak Ben Dhieb, Hatem Bouzaiene, Khaled Rahal

**Affiliations:** 1 Department of Surgical Oncology, Salah Azaiz Anti-cancer Institute, Tunis, Tunisia

**Keywords:** Breast cancer, risk factor, fibroadenoma, benign breast disease

## Abstract

Fibroadenomas are the most common breast disease that occurs usually in young. The coexistence of an invasive ductal carcinoma and a fibroadenoma in the ipsilateral breast is extremely rare. We present the case of a 52 years woman, presented to us for an upper-outer breast lump. Breast imaging concluded to tow contiguous lesions, one of them was suspicious. She had a conservative surgery. Histology concluded to a fibroadenoma and an invasive ductal carcinoma.

## Introduction

Breast cancer is the leading cause of mortality associated to female cancer among the world. Invasive ductal carcinoma is the most common pathological type. Fibroadenomas are the most common breast disease that occurs in women usually below the age of 35 years old [1]. A carcinoma can arise rarely within or in the ground of a fibroadenoma [2]. The coexistence of an invasive ductal carcinoma and a fibroadenoma in the ipsilateral breast is extremely rare which is illustrated in this case.

## Patient and observation

**Figure 1:**
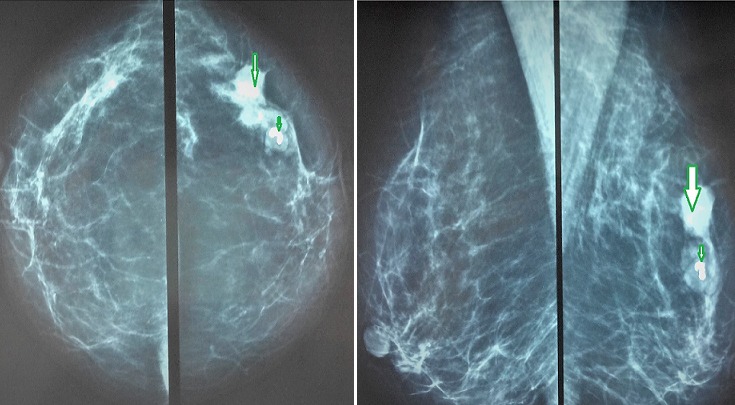
Bilateral mammography: A) craniocaudal view; B) mediolateral oblique view shows an ill-defined spiculated lesion of the right breast (large arrow) and an adjacent well defined opacity with central “popcorn” calcification (small arrow)

A 52 year-old woman presented with a mass located at the left breast. Clinical examination showed a 3cm palpable rough stiff lump located in the upper outer quadrant of the left breast and two left axillary lymph nodes. Breast ultrasound revealed a hypoechoic hyper-vascularized spiculated lesion which is “taller than broader” and measuring 26mm in diameter with posterior acoustic shadowing. This lesion was associated to another contiguous well-circumscribed, macrolobulated hypoechoic lesion measuring 27mm in diameter with intralesional calcification. Mammography showed a spiculated parenchymal distortion with an opaque center and thick spiculations, the second adjacent lesion was well-defined, isodense to the breast glandular tissue, with macrolobulation and central “coarse popcorn” calcifications evoking an aged fibroadenoma (Figure 1). No distant metastasis was detected on body CT scan. The patient was treated with breast conserving surgery. The lumpectomy measured 80 × 60 × 30mm. The histological examination concluded to a fibroadenoma and a contiguous 30mm diameter infiltrating ductal carcinoma SBR Grade III. Conservative surgery was performed with axillary lymph node dissection. The final histopathological report revealed atypical infiltrating carcinoma with grade SBR III. Three axillary lymph nodes were metastatic. ER and PR were negative on immunohistochemistry. HER2 was negative. The patient was referred for chemotherapy and radiotherapy.

## Discussion

Fibroadenoma is a biphasic tumor consisting of epithelial and stromal components [1]. Recent studies identified frequent mutations in the stromal component suggesting that this tumor was primarily stromal neoplasm [3]. Breast carcinoma may arise within a benign tumor or the tumors may coexist independently [3, 4]. In a review of the literature, the incidence of a carcinoma evolving within a fibroadenoma was 0.002% to 0.0125%. Only 10% of these tumors were infiltrating ductal carcinoma. The clinical and imaging findings are frequently those of benign fibroadenomas [5]. In our case, carcinomatous lesion was an infiltrating ductal carcinoma adjacent to an aged fibroadenoma. Researchers still debate about breast cancer risk and benign breast diseases. Fibroadenomas are not typically considered as a risk factor of carcinoma [6]. However, fibroadenoma shows in about half of the cases proliferative changes like sclerosing adenosis, epithelial calcifications and papillary apocrine metaplasia that classify it as complex fibroadenoma and a long term risk factor for breast cancer [1, 2]. The Mayo Clinic cohort study published in 2005 including 9087 women with benign breast lesions followed for 15 years found a relative risk of breast cancer at 1.56 (95% CI [1.45-1.68]) suggesting the presence of breast cancer precursors in benign breast diseases. After biopsy, the increased risk for developing a breast cancer persists for more than 25 years. Breast cancer was less important in the absence of proliferation and family history of breast cancer. In cases of proliferation, the presence of atypia was critical and family history was an independent risk factor for breast cancer [7].

A recent meta-analaysis conducted by Dytard et al of 32 papers, concluded to the existence of an increased risk in women with benign breast disease for developing breast cancer. The summary risk estimate of developing breast cancer following a biopsy showing non-proliferative disease was 1.17. It was 1.76 in case of proliferative disease without atypia [8]. These data were confirmed by a recent Mayo Clinical study published in 2015 that analyzed the risk of breast cancer in 9076 women aged 18 to 65 who had a breast biopsy or a breast lesion excision, among which 20.2% had simple fibroadenomas and 3.3% had complex fibroadenomas. Compared to patients without fibroadenoma (RR = 1.51, 95% CI [1.40-1.63]), the risk of breast cancer was not increased in patients with single fibroadenoma (RR = 1.49, 95% CI [1.26-1.74]) while there was a significant increase in patients with complex fibroadenoma (RR = 2.27, 95% CI [1.63-3.10]). The authors have demonstrated the importance of proliferative character with or without atypia in the adjacent parenchyma [9]. The existence of proliferative changes in the parenchyma adjacent to the fibroadenoma elevates further the risk [5]. A study on 3567 women diagnosed with breast cancer published in April 2018 by Nutter *et al* found that woman with breast proliferative disease with atypia are twice exposed to have multifocal breast cancer than woman with no breast benign disease [10].

## Conclusion

This case highlights the rare association in contiguity of two common breast diseases and the value of histological examination for the diagnosis of malignancy. Every woman with the diagnosis of a complex fibroadenoma particularly with a family history is more concerned by frequent mammographic screening starting at an early age.

## Competing interests

The authors declare no competing interests.
